# Exploring the Role of Artificial Intelligence (AI)-Driven Training in Laparoscopic Suturing: A Systematic Review of Skills Mastery, Retention, and Clinical Performance in Surgical Education

**DOI:** 10.3390/healthcare13050571

**Published:** 2025-03-06

**Authors:** Chidozie N. Ogbonnaya, Shizhou Li, Changshi Tang, Baobing Zhang, Paul Sullivan, Mustafa Suphi Erden, Benjie Tang

**Affiliations:** 1 Surgical Skills Centre, Dundee Institute for Healthcare Simulation, Respiratory Medicine and Gastroenterology, School of Medicine, Ninewells Hospital and Medical School, University of Dundee, Dundee DD1 9SY, UK; 2 Hammersmith Hospital, Hammersmith Campus, Imperial College, London W12 0HS, UK; 3 School of Medicine, University of Edinburgh, Edinburgh EH8 9YL, UK; 4 School of Engineering and Physical Sciences, Heriot Watt University, Edinburgh EH14 4AS, UK; b.zhang@hw.ac.uk (B.Z.);

**Keywords:** laparoscopic suturing, artificial intelligence, laparoscopic skill acquisition, laparoscopic training, surgical performance, surgical education

## Abstract

**Background:** Artificial Intelligence (AI)-driven training systems are becoming increasingly important in surgical education, particularly in the context of laparoscopic suturing. This systematic review aims to assess the impact of AI on skill acquisition, long-term retention, and clinical performance, with a specific focus on the types of machine learning (ML) techniques applied to laparoscopic suturing training and their associated advantages and limitations. **Methods:** A comprehensive search was conducted across multiple databases, including PubMed, IEEE Xplore, Cochrane Library, and ScienceDirect, for studies published between 2005 and 2024. Following the PRISMA guidelines, 1200 articles were initially screened, and 33 studies met the inclusion criteria. This review specifically focuses on ML techniques such as deep learning, motion capture, and video segmentation and their application in laparoscopic suturing training. The quality of the included studies was assessed, considering factors such as sample size, follow-up duration, and potential biases. **Results:** AI-based training systems have shown notable improvements in the laparoscopic suturing process, offering clear advantages over traditional methods. These systems enhance precision, efficiency, and long-term retention of key suturing skills. The use of personalized feedback and real-time performance tracking allows learners to gain proficiency more rapidly and ensures that skills are retained over time. These technologies are particularly beneficial for novice surgeons and provide valuable support in resource-limited settings, where access to expert instructors and advanced equipment may be scarce. Key machine learning techniques, including deep learning, motion capture, and video segmentation, have significantly improved specific suturing tasks, such as needle manipulation, insertion techniques, knot tying, and grip control, all of which are critical to mastering laparoscopic suturing. **Conclusions:** AI-driven training tools are reshaping laparoscopic suturing education by improving skill acquisition, providing real-time feedback, and enhancing long-term retention. Deep learning, motion capture, and video segmentation techniques have proven most effective in refining suturing tasks such as needle manipulation and knot tying. While AI offers significant advantages, limitations in accuracy, scalability, and integration remain. Further research, particularly large-scale, high-quality studies, is necessary to refine these tools and ensure their effective implementation in real-world clinical settings.

## 1. Introduction

Laparoscopic surgery, also known as minimally invasive surgery, involves making small incisions that are typically 0.5 to 1.5 cm in the abdomen, through which surgical instruments and a laparoscope (a thin tube with a camera and light) are inserted to provide a view of the abdominal or pelvic cavity during procedures [1,2,3,4]. Among the various skills required for laparoscopic procedures, laparoscopic suturing is particularly crucial. This skill has emerged as a cornerstone in modern minimally invasive surgery, representing a significant advancement over traditional open techniques [5]. Laparoscopic suturing enables surgeons to perform complex operations with high precision through small incisions and specialized instruments. The benefits of laparoscopic surgery, including reduced recovery times, minimized postoperative pain, and a lower risk of complications compared to open surgery, are well-documented [6,7]. However, mastering laparoscopic suturing is one of the most challenging aspects of surgical training. This difficulty arises from factors such as limited dexterity with laparoscopic instruments, restricted visual fields that impair spatial awareness, and the complexities of indirect instrument handling [8,9,10]. Consequently, the effective training of surgical trainees in laparoscopic suturing techniques is of paramount importance to ensure their preparedness for the demanding realities of the operating room.

When it is well recognized that practicing and acquiring laparoscopic suturing skills in real surgical procedures are practically difficult and ethically wrong, traditionally, laparoscopic suturing has predominantly relied on mentorship-based approaches. These methods often utilize physical models, cadavers, or direct observation of live surgical procedures, fostering a hands-on learning environment. While these conventional techniques have historically served as the cornerstone of surgical education, they frequently fall short in adequately preparing trainees for the complexities they will encounter in real-world surgical scenarios. Limitations such as the ethical constraints surrounding the use of cadaveric models, the high costs associated with animal models, and the lack of realism in synthetic simulators have raised concerns about the effectiveness and accessibility of traditional training methods [11,12,13]. This limitation underscores the urgent need for innovative training solutions that can significantly enhance skill acquisition and retention, ultimately leading to improved surgical outcomes.

Recent advancements in Artificial Intelligence (AI) and related technologies are transforming surgical education, particularly in laparoscopic suturing [14]. AI, which involves simulating human intelligence in machines, enables real-time feedback, personalized learning, and data-driven performance analytics, addressing many limitations of traditional training methods [15,16,17]. Virtual reality (VR), which creates immersive, computer-generated environments, plays a key role in enhancing these training experiences. The relationship between AI and VR lies in their combination. AI enhances VR by providing real-time performance analytics, adaptive feedback, and error detection, creating a highly realistic and individualized learning environment. This combination allows for more effective and tailored training that prepares trainees for real-world clinical challenges [18].

As laparoscopic surgery continues to gain prominence within modern surgical practice, providing minimally invasive options for a wide range of procedures, the importance of developing effective training methods cannot be overstated. The demand for skilled laparoscopic surgeons is increasing, making it crucial to ensure that surgical trainees achieve a high level of competency in these techniques [16,19]. However, current literature indicates a significant gap in understanding how AI-enhanced training systems can be integrated into surgical education and their impact on long-term skill retention and clinical outcomes [20,21].

This systematic review aims to provide a comprehensive synthesis of current knowledge regarding AI-enhanced training systems for laparoscopic suturing. By examining the application of AI in this specific area of training, the review will establish a foundation for future advancements. Additionally, it will assess the effectiveness of AI-based training in promoting skill acquisition, retention, and clinical performance among surgical trainees, thereby contributing to the ongoing conversation in the field of surgical education.

## 2. Methodology

### 2.1. Search Strategy and Databases

To ensure a comprehensive search, we employed the databases PubMed, IEEE Xplore, Cochrane Library, and ScienceDirect. The search strategy involved combining terms and Boolean operators such as “Laparoscopic training AND AI OR machine learning”, “Surgical education AND virtual reality AND laparoscopic suturing”, and “AI feedback OR real-time feedback in surgery”. Each database query was limited to studies published from 2005 to 2024 in English to capture recent advancements in AI and VR applications. The review was registered with Open Science Framework (osf.io/c2pm9).

### 2.2. Inclusion and Exclusion Criteria

The inclusion criteria consisted of studies assessing AI-based laparoscopic suturing training for skill acquisition or retention, comparison studies between AI-enhanced and traditional training methods, and studies evaluating outcomes such as precision, time efficiency, and overall performance improvement. The exclusion criteria included studies that did not focus on laparoscopic suturing (e.g., unrelated surgical techniques), non-peer-reviewed papers, commentaries, or opinion pieces, and studies focusing solely on robotic surgical methods without AI-driven training components.

### 2.3. Study Selection and Screening Process

**Figure 1:**
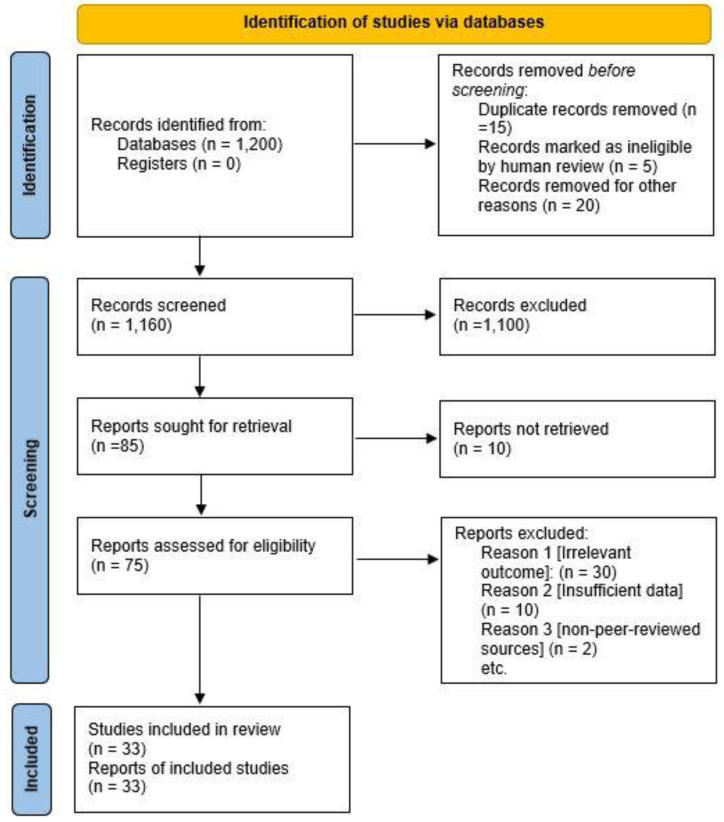
PRISMA flow diagram of study selection.

An initial search yielded 1200 potential studies, which were screened by title and abstract. After removing duplicates and applying inclusion criteria, 85 articles were selected for full-text review. Of these, 33 studies met the full inclusion criteria and were included in this review (Figure 1). This systematic review was conducted in accordance with the Preferred Reporting Items for Systematic Reviews and Meta-Analyses (PRISMA) guidelines to ensure transparency, reproducibility, and rigor in reporting the methodology and results [22]. The entire process, including study selection, screening, and data extraction, was carried out by human researchers to ensure accuracy and adherence to the guidelines.

### 2.4. Data Extraction

The following data were extracted from each included study: author(s) and year, study design such as randomized control trial or cohort study, sample size, type of intervention including AI model type, VR platform, or feedback system, outcome measures such as accuracy, time efficiency, and skill retention, and the main findings.

### 2.5. Risk of Bias Assessment

The Cochrane Risk of Bias (RoB) Tool and PRISMA guidelines were used to assess the quality of the studies. The assessment focused on potential sources of bias, including selection bias by ensuring participant randomization in controlled studies, performance bias by comparing interventions under matched conditions, detection bias by ensuring consistency in outcome assessment, and attrition bias by accounting for participant dropout rates, particularly in longitudinal studies.

## 3. Results and Findings

Table 1 summarizes the 33 studies included in this systematic review, which evaluated the effectiveness of AI-assisted tools and traditional methods in training surgical skills, with a particular focus on laparoscopic suturing. These studies employed various research designs, including randomized controlled trials (RCTs), nonrandomized studies, and experimental designs, conducted across diverse global locations.

A significant number of the studies demonstrated the effectiveness of AI-enhanced feedback in improving surgical skills. For example, Ma et al. (2023) [23] reported that AI-based video feedback led to substantial improvements in needle handling, especially for underperforming trainees, underscoring the potential of AI to enhance skill acquisition in laparoscopic tasks. Similarly, Laca et al. (2023) [45] found that AI feedback in robotic suturing tasks was particularly beneficial for novice trainees, emphasizing the importance of personalized feedback in optimizing skill development. These studies provide valuable insights into the role of AI-assisted tools in laparoscopic suturing training, highlighting both the potential and challenges of integrating AI into surgical education.

One notable outcome was the effectiveness of AI-driven feedback in improving skill acquisition. Ma et al. (2023) [23] demonstrated that AI-based video feedback significantly improved needle handling, particularly among underperforming trainees, suggesting that AI can be a powerful tool for developing suturing skills. In a similar vein, Naik et al. (2017) [24] found that personalized video feedback resulted in faster performance and higher completion rates for novices, although it did not significantly impact OSATS scores. This indicates that while AI feedback may accelerate learning speed, it might not fully capture all aspects of surgical proficiency, especially those evaluated by standardized rating scales.

The impact of different types of feedback mechanisms also emerged as a key theme. Huang et al. (2024) [25] demonstrated that force-feedback systems helped reduce the learning curve for novices by improving grip control, facilitating both immediate skill acquisition and retention. Montoya-Alvarez et al. (2024) [28] also found that visual feedback in laparoscopic suturing enhanced proficiency and shortened the learning curve more effectively than haptic feedback or no feedback at all. This suggests that visual feedback may be especially effective in efficiently developing suturing skills.

AI’s potential to make training more accessible, particularly in low-resource settings, was another important finding. Ryder et al. (2023) [27] demonstrated that AI-based scoring systems accurately matched human assessments in countries such as Ethiopia and Cameroon, showing how AI can bridge the training gaps in resource-limited environments. Alonso-Silverio et al. (2018) [35] further highlighted the cost-effectiveness and scalability of AI-powered training tools, making them particularly beneficial in regions with limited access to expert instructors.

Real-time evaluations and personalized feedback were also emphasized as key advantages of AI-driven training tools. Mohaidat et al. (2024) [31] utilized deep learning models to assess peg transfer and suturing tasks with high precision, providing real-time feedback that allowed trainees to adjust their techniques immediately. This personalized guidance accelerated skill development and retention, making it an effective learning tool for surgical trainees.

AI was also found to provide more standardized and objective assessments. Perumalla et al. (2022) [51] applied video segmentation algorithms to identify key surgical maneuvers such as suturing and knot tying. Their results demonstrated that AI was highly accurate in differentiating between these steps, offering a more objective assessment of skills that reduces the variability commonly present in human evaluations. Furthermore, Ko et al. (2017) [39] showed that AI’s objective evaluations of suturing tasks helped trainees better assess their own skills, although novices still required more time to master the techniques.

In clinical settings, AI demonstrated its potential to enhance decision-making and surgical performance. Zuluaga et al. (2024) [32] showed that AI models could predict errors during robotic suturing, providing real-time guidance to surgeons, which could potentially improve patient safety and surgical outcomes.

AI also contributed to increased training efficiency. Bogar et al. (2024) [18] reported that AI in virtual reality simulators reduced assessment time by 30% while maintaining 95% accuracy. This increased efficiency is particularly important in settings with limited access to instructors, as it allows for more training to take place in a shorter amount of time.

Several studies explored the application of primary machine learning (ML) techniques in laparoscopic suturing training. These techniques, particularly deep learning algorithms and computer vision, were used to assess surgical performance and provide real-time feedback. For example, Mohaidat et al. (2024) [31] employed deep learning models to assess peg transfer and suturing tasks, achieving high precision in evaluating trainee performance. These ML systems demonstrated the potential to standardize assessments, making them more objective and reducing the reliance on human evaluators. Similarly, Perumalla et al. (2022) [51] applied deep learning algorithms for video segmentation and procedural step identification, achieving high accuracy in differentiating basic maneuvers such as suturing, knot tying, and suture cutting. These applications emphasize the growing role of machine learning in enhancing the precision and efficiency of laparoscopic suturing training, enabling more accurate and consistent assessments. Which underscores the transformative potential of AI-driven tools in laparoscopic suturing training. AI and ML techniques not only improve skill acquisition, retention, and clinical performance, but also enhance accessibility, efficiency, and objectivity in surgical education. Despite challenges related to the accuracy of AI in complex real-world scenarios, these technologies show great promise in advancing surgical training and improving patient outcomes.

**Figure 2:**
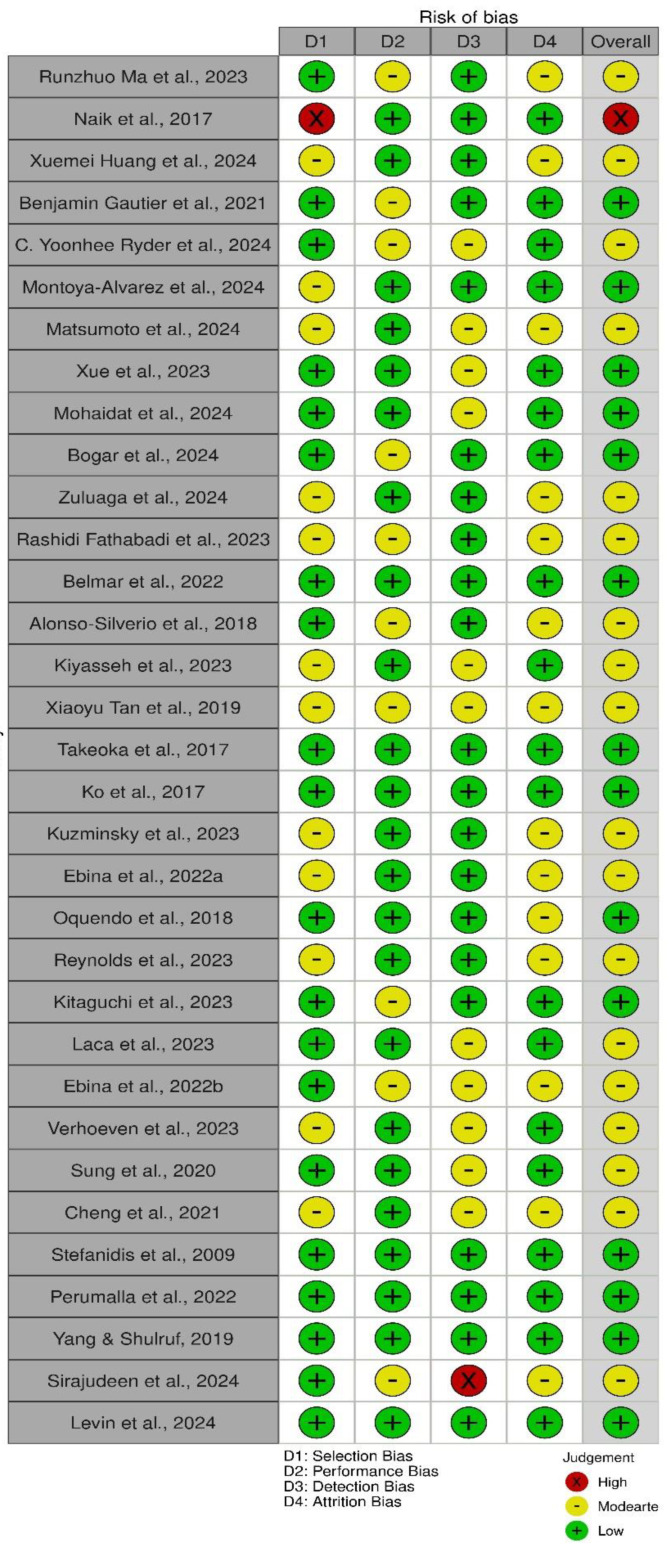
Risk of bias assessment for all 33 studies [18,23,24,25,26,27,28,29,30,31,32,33,34,35,36,37,38,39,40,41,42,43,44,45,46,47,48,49,50,51,52,53,54].

The risk of bias assessment for the included studies is presented in Figure 2. This evaluation examined four types of bias: selection bias, performance bias, detection bias, and attrition bias, with an overall bias rating assigned for each study.

A substantial portion of the studies demonstrated a low risk of bias across multiple categories, indicating a high level of reliability in the evidence. For example, Ma et al. (2023) [23], Ko et al. (2017) [39], and Levin et al. (2024) [54] exhibited low risk in selection, performance, and detection biases, supporting the validity of their findings. These studies contribute to the growing body of evidence supporting the effectiveness of AI-driven systems in improving surgical skills (Figure 2).

However, several studies were classified as having moderate risk of bias, largely due to non-randomized designs or partial blinding. For example, Montoya-Alvarez et al. (2024) [28] and Fathabadi et al. (2023) [33] showed moderate performance bias and selection bias. While these studies still provide valuable insights into the potential benefits of AI feedback and other interventions, the risk of bias should be considered when interpreting their results.

**Figure 3:**
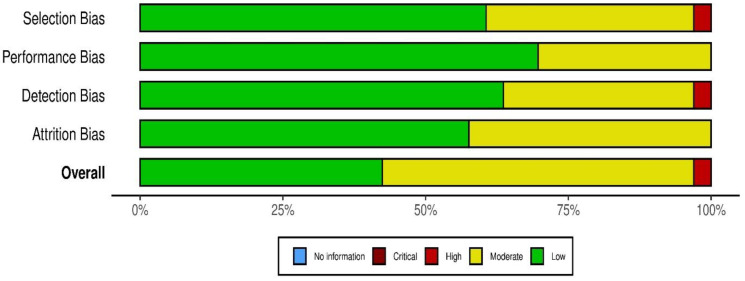
Summary of risk of bias across bias domains [18,23,24,25,26,27,28,29,30,31,32,33,34,35,36,37,38,39,40,41,42,43,44,45,46,47,48,49,50,51,52,53,54].

A few studies were assessed as having a high risk of bias, particularly due to issues with selection or detection biases (Figure 3). Naik et al. (2017) [24] and Sirajudeen et al. (2024) [53] were rated as having high risk in certain categories, primarily due to the lack of randomized control or insufficient blinding in their study designs. These studies highlight the need for future research employing more rigorous methodologies, such as randomized controlled trials, to further validate the effectiveness of AI-based interventions in surgical training. While the studies included in this review provide promising evidence for the use of AI in surgical education, the presence of moderate to high risk of bias in some studies calls for caution in the interpretation of results. These findings underscore the need for more robust research designs, particularly RCTs, in future studies to strengthen the evidence base for AI’s role in surgical training.

## 4. Discussion

This systematic review examines the growing influence of AI in surgical training, with a particular focus on laparoscopic suturing, a crucial yet often neglected skill in modern surgery. Drawing on 33 studies, the review highlights how AI-powered training systems are transforming surgical education. While past research has generally explored AI’s impact on broader surgical skills, this review specifically focuses on suturing, a vital skill that is difficult to master yet often overlooked. Importantly, the review goes beyond evaluating short-term performance by considering both skill acquisition and long-term retention, which are critical for maintaining competence in suturing as the field of surgery evolves.

The primary machine learning (ML) techniques applied in laparoscopic suturing training include deep learning models, video segmentation, motion tracking, and reinforcement learning. These approaches offer several important benefits: they provide real-time, personalized feedback, which helps trainees improve suturing skills like needle handling, knot tying, and motion control more efficiently Naik et al. (2017) [23], Montoya-Alvarez et al. (2024) [27]. Deep learning models, in particular, have demonstrated high accuracy in assessing complex suturing tasks and differentiating skill levels, while video segmentation breaks down procedural steps to provide more detailed feedback Perumalla et al. (2022) [50]. Motion tracking and force-feedback technologies have enhanced grip control and reduced the learning curve for novices, making training more accessible, particularly in resource-limited settings Xuemei Huang et al. (2024) [24], Alonso-Silverio et al. (2018) [34]. Furthermore, reinforcement learning enables tailored feedback and improves decision-making, which further advances skill development Xiaoyu Tan et al., (2019) [36]. These AI-driven innovations ultimately enhance training efficiency, accuracy, and scalability Bogar et al. (2024) [31]. The segmentation accuracy in suturing tasks is heavily influenced by the choice of algorithms, with deep learning methods often outperforming traditional machine learning approaches. For example, Mohaidat et al. (2024) [31] achieved a high segmentation accuracy of 92% using deep learning models like Convolutional Neural Networks (CNNs) and U-Net, which excel at automatically extracting complex features from large datasets. This allowed their model to adapt to intricate suturing patterns and achieve precise segmentation. In contrast, Perumalla et al. (2022) [51] used traditional machine learning methods, such as Support Vector Machines (SVM) and Random Forests, which rely on manually crafted features like texture and edge detection. Although effective in simpler tasks, these algorithms fell short in capturing the complex variations present in suturing, leading to a lower accuracy of 84%. The differences in dataset quality also played a significant role, as Mohaidat et al. (2024) [31] used a high-resolution, domain-specific dataset that enabled better feature extraction, while Perumalla et al. (2022) [51] worked with a more general dataset that lacked the fine details needed for more accurate segmentation. To further improve AI in suturing, several strategies could be considered. Expanding the dataset to include more diverse suturing patterns and surgical images from various clinical settings would help the model generalize better. Combining traditional machine learning with deep learning techniques could also enhance performance, where initial feature extraction by traditional methods could be refined by deep learning models. Moreover, utilizing transfer learning, where models pre-trained on larger, related datasets are fine-tuned for suturing, could significantly boost accuracy.

One of the major contributions of this review is the identification of the key AI methods most commonly used across the studies reviewed. These include real-time feedback systems, deep learning models, motion capture analysis, and video segmentation algorithms. For example, studies by Naik et al. (2017) [23], Montoya-Alvarez et al. (2024) [27], and Laca et al. (2023) [44] demonstrated the effectiveness of AI-based video feedback in improving suturing skills, particularly for underperforming trainees. In addition, deep learning models, as employed by Mohaidat et al. (2024) [30] and Perumalla et al. (2023) [50], offered precise assessments of suturing tasks, contributing to the standardization of skill evaluations. The review also discusses how AI can enhance different stages of the suturing process. For instance, needle manipulation was significantly improved through real-time video feedback and algorithmic analysis, as shown by Laca et al. (2023) [44]. Insertion and pulling techniques were refined by assessing trajectory and tissue handling efficiency, as demonstrated by Runzhuo Ma et al. (2024) [22]. Knot tying was improved using AI-assisted systems that ensured consistent tension, as noted by Montoya-Alvarez et al. (2024) [27]. Grip control was enhanced through force-feedback-enabled tools, effectively shortening the learning curve for novice surgeons, as evidenced by Xuemei Huang et al. (2024) [24]. While force-feedback systems are essential, it is believed that haptic feedback should also be considered crucial, especially for newcomers in laparoscopic surgery. Haptic feedback provides tactile sensations that simulate the feel of real tissue, improving a surgeon’s ability to navigate and manipulate instruments, making it an important tool for skill acquisition [55]. In addition to haptic feedback, visual feedback plays a vital role in enhancing proficiency and shortening the learning curve in laparoscopic suturing. Studies have shown that visual feedback, where trainees receive real-time visual cues about their technique, has proven more effective than no feedback at all [56,57,58]. This suggests that incorporating visual feedback into training systems could be particularly beneficial in accelerating the development of suturing skills.

Several studies have also highlighted the potential of AI in robotic surgery. Runzhuo Ma et al. (2024) [23] showed that AI-based video feedback significantly enhanced needle handling in underperforming surgeons, leading to improvements in suturing performance. Similarly, Laca et al. (2023) [45] found that AI feedback improved both needle handling and driving skills during robotic suturing, with the most notable benefits seen in novice surgeons. These findings suggest that AI-driven feedback can be personalized to match an individual’s skill level, optimizing the learning process and accelerating proficiency. In laparoscopic surgery, which demands precision and real-time decision-making, AI has demonstrated substantial promise. Ryder et al. (2024) [27] Ryder et al. (2024) [27] reported that AI-based scoring of laparoscopic simulation videos showed a 72.4% agreement rate with human assessments, which underscores AI’s potential to assist in surgical training, particularly in areas where expert trainers are scarce. However, challenges remain in terms of the high cost of AI, virtual reality (VR), and machine learning technologies, which may limit their accessibility, particularly in low- and middle-income countries. While AI and VR hold promises, there may still be a significant gap in affordability for these devices to become widespread solutions for surgeon training in these regions. Moreover, the drop in acquisition costs for these devices will be crucial to their success as an efficient training tool. Although AI can improve training efficiency and provide valuable feedback, it is important to acknowledge that traditional hands-on training on living tissue, which is still more affordable in some regions, may continue to be a more practical solution for certain environments, despite the growing role of AI. Bogar et al. (2024) [18] found that AI-driven virtual reality (VR) simulators were as effective as traditional box trainers in laparoscopic training, while reducing assessment time by 30%, without compromising accuracy. Montoya-Alvarez et al. (2024) [28] highlighted that real-time visual feedback outperformed both haptic feedback and no feedback in enhancing laparoscopic suturing skills. Furthermore, Matsumoto et al. [29] used AI-assisted kinematic analysis to demonstrate significant performance differences between novice and expert surgeons during gastrectomy procedures, illustrating AI’s potential for both training and real-time evaluation. Automated performance metrics have significantly evolved in both robotic and non-robotic AI-assisted surgery, transforming skill assessment from subjective expert evaluations to objective, data-driven analysis. Historically, structured training programs, such as those studied by Stefanidis et al. (2009) [50], established the foundation for standardized performance evaluation in laparoscopic suturing. Over time, AI-based techniques, including motion tracking and deep learning, have enhanced these assessments by providing real-time feedback. In robotic surgery, studies like Sirajudeen et al. (2024) [53] demonstrated AI’s capability to predict surgical errors with accuracy comparable to expert assessments, while Levin et al. (2024) [54] highlighted AI’s ability to identify key procedural steps in robotic-assisted hysterectomy. Similarly, in non-robotic laparoscopy, research by Xue et al. (2023) [30] showed AI’s effectiveness in pass/fail evaluation of laparoscopic skills, achieving 78.6% accuracy in real-time assessment. Additionally, Perumalla et al. (2022) [51] reported AI segmentation achieving 84% accuracy in differentiating key suturing maneuvers, further supporting automated skill evaluation.

Machine learning (ML) models have been widely used to automate the assessment of surgical skills, which have traditionally relied on subjective human evaluation. Kowalewski et al. (2019) [59]. applied ML algorithms to assess suturing skills, achieving 82.2% accuracy in correctly identifying beginner skill levels. This study utilized data captured by Myo armbands to monitor key motion metrics such as acceleration, angular velocity, and orientation. The ensemble of binary-class neural networks demonstrated the highest accuracy for skill level classification, particularly distinguishing between beginners, intermediates, and experts. This finding underscores the potential of AI and machine learning to provide objective, reliable, and automated proficiency assessments in surgical training, offering a more standardized and efficient alternative to traditional expert evaluations. This study also highlights the role of modern technology, such as Dynamic Time Warping (DTW), in enhancing the analysis of surgical motion data for phase detection and workflow analysis. Kiyasseh et al. (2023) [36] found that AI feedback in video-based evaluations aligned closely with human expert assessments, providing consistent and reproducible feedback. In credentialing, Ebina et al. (2022) [41] demonstrated that AI, combined with motion capture technology, enabled objective skill assessments in wet lab training, offering a scalable and consistent approach for credentialing across institutions. Mohaidat et al. (2024) [31] showed that deep learning models applied to peg transfer and suturing tasks achieved high precision, underscoring AI’s potential for standardizing training evaluations. Beyond training, AI has shown promise in real-time decision-making during live surgeries. Zuluaga et al. (2024) [32] demonstrated how AI-powered annotations during robotic surgeries could highlight critical steps and safety milestones, assisting surgeons in key intraoperative decisions. Dayan et al. (2024) [60] explored AI’s role in evaluating laparoscopic appendectomy, where the AI system assessed surgical complexity and safety but did not correlate strongly with postoperative outcomes. This suggests that while AI excels in real-time assessments, further refinement is required to link AI evaluations with clinical results.

AI-powered training tools have proven especially beneficial in resource-limited settings. Alonso-Silverio et al. (2018) [35] highlighted how an affordable AI-powered laparoscopic trainer could improve accessibility and build confidence in skill acquisition, especially in areas lacking expert surgeons and advanced training tools. Reynolds et al. (2023) [43] demonstrated that the APPY-VOP tool could accurately differentiate skill levels in laparoscopic surgical training, offering a scalable solution for training in LMICs. Xue et al. (2023) [30] developed an AI-powered pass/fail evaluation system for the Fundamentals of Surgery (FLS), which achieved a 78.6% accuracy in real-time assessments, streamlining training by providing more efficient feedback. Cheng et al. (2022) [49] used deep learning to analyze laparoscopic cholecystectomy procedures, where AI demonstrated high accuracy in identifying surgical phases, though performance varied with case complexity, indicating that further optimization is needed for more advanced tasks. Sung et al. (2020) [48] and Xuemei Huang et al. (2024) [25] explored AI-assisted haptic feedback systems in virtual simulations, finding that combining AI with haptic feedback reduced training time and costs while enhancing learning experiences, particularly for novices mastering complex skills like suturing and laparoscopic handling.

Despite the promising advancements in AI for suturing training, several challenges and limitations remain. While instrument tracking has shown improvements in suturing skills, the weak correlation between AI assessments and expert evaluations indicates the need for further refinement to enhance reliability. Although deep learning models can predict errors in robotic suturing, real-world complexities such as patient-specific factors and surgical nuances still require further development. AI and machine learning technologies hold great promise in advancing suturing training by offering personalized feedback, improving skill assessments, and supporting real-time decision-making. However, challenges persist in terms of accuracy, scalability, and generalizability. The integration of these systems into clinical practice also faces obstacles, including the need for high-quality data, specialized equipment, and overcoming cost and infrastructure barriers. Ethical concerns surrounding data privacy and AI-driven decision regulation must be addressed. The variability in research quality and the difficulties in applying AI to various suturing techniques further emphasize the need for larger, higher-quality studies. With continued refinement, AI has the potential to become a vital tool in suturing training, offering both immediate and long-term benefits to healthcare systems worldwide.

## 5. Conclusions

This review explores the growing use of Artificial Intelligence in laparoscopic suturing training, highlighting the key machine learning techniques like deep learning, motion capture, and video segmentation that are improving essential skills such as needle handling, knot tying, and grip control. These AI tools provide real-time feedback, allowing trainees to develop their skills more effectively and consistently. Beyond just helping with technical proficiency, AI also supports better decision-making during surgery, which ultimately improves the quality of procedures and enhances patient safety. One of the key advantages of AI-driven training tools is their capacity to provide real-time evaluation of a surgeon’s performance, which can significantly enhance training efficiency. While AI has demonstrated substantial promise in improving surgical education, its ability to make training more accessible, especially in areas with limited resources and fewer expert instructors, remains uncertain. While AI has the potential to bridge some gaps, challenges such as accuracy variations and the high cost of implementation still need to be addressed for it to be truly effective in resource-limited settings. However, challenges remain, such as variations in AI accuracy, particularly in complex, real-world situations. Despite these hurdles, the continued development of AI holds the potential to transform suturing training, making it more efficient, scalable, and capable of improving surgical outcomes worldwide.

## Figures and Tables

**Table 1:** Summary of all 33 included studies.

Author(s), Year	Title	Research Design	Study Location	Key Findings
Runzhuo Ma et al., 2023 [23]	AI-based Video Feedback on Robotic Suturing Tasks	Randomized Controlled Trial	United States of America (USA)	AI feedback improved needle handling in underperformers; significant but selective performance improvement for suturing skill acquisition.
Naik et al., 2017 [24]	Personalized video feedback improves suturing skills of incoming general surgery trainees	Nonrandomized study: video feedback vs. no feedback	USA	The feedback group had higher completion rates and faster performance. Significant improvement in suturing time for the feedback group. Higher global ratings for feedback group. No difference in OSATS scores.
Xuemei Huang et al., 2024 [25]	Force-Feedback-Enabled Laparoscopic Grasper for Skill Improvement	Pre-experiment Training Program	China	Force feedback reduced the learning curve for novices and improved grip control.
Benjamin Gautier et al., 2021 [26]	Real-Time 3D Tracking System for Instrument Feedback in Laparoscopy	Algorithm Development	United Kingdom (UK)	Cost-effective, automated feedback system preserving physical interaction during training.
Ryder et al., 2023 [27]	Using Artificial Intelligence to Gauge Competency on a Novel Laparoscopic Training System	Experimental Study	Ethiopia, Cameroon, Kenya, and USA	AI-based scoring of laparoscopic simulation videos matched human assessments ~72.4% of the time. AI can help address training gaps in LMICs.
Montoya-Alvarez et al., 2024 [28]	Real-Time Feedback’s Impact on Laparoscopic Suturing Skills	Randomized Controlled Trial	Mexico	Visual feedback enhanced suturing proficiency and shortened the learning curve compared to haptic feedback or no feedback.
Matsumoto et al., 2024 [29]	Kinematic Analysis of Gastrectomy Using AI	Retrospective Video Analysis	Japan	AI revealed differences in efficiency between novice and expert surgeons, suggesting application for real-time evaluation.
Xue et al., 2023 [30]	Pass/Fail Evaluation System for Fundamentals of Surgery (FLS)	Experimental Video Analysis	USA	AI achieved 78.6% accuracy in real-time assessment, improving feedback and training efficiency.
Mohaidat et al., 2024 [31]	AI-Driven Deep Learning for Peg Transfer and Suturing Assessment	Developmental Study with Computer Vision	USA (Multiple Centers)	Deep learning models provided high precision in peg transfer tasks, offering potential for standardized training evaluation.
Bogar et al., 2024 [18]	Comparison of VR Simulators vs. Box Trainers for Laparoscopic Training	Randomized Controlled Study	Hungary	VR simulators were equally effective as box trainers; AI reduced assessment time by 30%, maintaining 95% accuracy.
Zuluaga et al., 2024 [32]	Real-Time AI Annotations for Robotic Surgery	Symposium and Live Demonstration	Palo Alto, USA	AI annotated key steps and safety milestones during live surgeries, demonstrating potential for intraoperative decision-making.
Rashidi Fathabadi et al., 2023 [33]	Multi-Camera AI-Based Assessment for Peg Transfer Tasks	Experimental Study with Deep Learning	USA	High-precision assessment model for peg transfer tasks, eliminating the need for manual evaluation.
Belmar et al., 2023 [34]	AI-Expert Agreement on Remote Training Platforms	Observational Study	Latin America	AI showed 93% agreement with expert evaluations, indicating reliability for large-scale training assessment.
Alonso-Silverio et al., 2018 [35]	Affordable AI-Powered Laparoscopic Trainer	Experimental Design	Mexico	The AI-powered system improved accessibility and confidence in skill acquisition in resource-limited settings.
Kiyasseh et al., 2023 [36]	Reliability of AI Feedback in Surgical Video Explanations	Multi-Institutional AI Feedback Study	Multiple Locations	AI feedback often aligned with human evaluations, with improvements via explanation strategies like TWIX.
Xiaoyu Tan et al., 2019 [37]	Robot-Assisted Training System with Deep Reinforcement Learning	Experimental Robot-Assisted Training Study	Singapore	Reinforcement learning significantly improved laparoscopic training outcomes, providing tailored feedback and action simulation.
Takeoka et al., 2017 [38]	Assessment potential of a new suture simulator in laparoscopic surgical skills training	Pre- and post-training evaluation using a hybrid simulator	Japan	Significant improvement in air pressure leakage, number of full-thickness sutures, suture tension, wound area, and performance time. Performance was significantly better post-training.
Ko et al., 2017 [39]	Randomized Controlled Trial Comparing Trainee-Directed Virtual Reality Simulation Training and Box Trainer on the Acquisition of Laparoscopic Suturing Skills	Randomized Controlled Trial (RCT)	China	No significant difference in successful task completion, suturing time, or assessment scores between virtual reality simulator, box trainer, and control groups. Novices required longer training time to master suturing. Trainees had difficulty accurately assessing their own skill level.
Kuzminsky et al., 2023 [40]	Comparing AI and Traditional Assessments in Veterinary Suturing Skills	Comparative Observational Study	USA	AI showed higher reliability in skill differentiation, suggesting potential for objective assessment in surgical education.
Ebina et al., 2022 [41]	AI for Objective Surgical Skill Assessment in Wet Lab Training	Experimental Study with Motion Analysis	Japan	AI and motion capture metrics correlated with expert ratings, demonstrating potential for automated skill credentialing.
Oquendo et al., 2018 [42]	Paediatrics Laparoscopic Suturing Evaluation with AI	Experimental Study with Motion Tracking	USA	AI achieved 71% alignment with human expert ratings, enhancing feedback for pediatric surgical training.
Reynolds et al., 2023 [43]	Evidence Supporting Performance Measures of Laparoscopic Appendectomy Through a Novel Surgical Proficiency Assessment Tool and Low-Cost Laparoscopic Training System	Pilot Study	Ethiopia, Cameroon, and USA	The APPY-VOP tool effectively distinguished skill levels in laparoscopic appendectomy. Peer rating showed consistent evaluations. Promising for training scalability.
Kitaguchi et al., 2023 [44]	Suturing Skill Assessment in Trans-anal Total Meso-rectal Excision Using Deep Learning	Retrospective Video Analysis Study	Japan	AI scoring correlated with manual assessments, supporting its use in skill evaluation for advanced surgical tasks.
Laca et al., 2023 [45]	AI-Based Video Feedback to Improve Novice Performance on a Robotic Suturing Task	Randomized Controlled Trial (RCT)	USA	AI-based feedback improved needle handling and driving skills, with underperformers benefiting the most, demonstrating the potential of personalized AI feedback in training.
Ebina et al., 2022 [46]	Machine Learning Credentialing System for Porcine Organ Simulations	Experimental Study	Japan	Machine learning demonstrated 70% accuracy in assessing laparoscopic competence, supporting scalable skill assessments.
Verhoeven et al., 2022 [47]	Assessment of Minimally Invasive Suturing Skills	Experimental Study	Netherlands	Instrument tracking showed skill improvement but had weak correlation with expert assessments.
Sung et al., 2020 [48]	Intelligent Haptic Virtual Simulation for Suture Surgery	Experimental Study	Republic of Korea	Developed an AI-assisted haptic VR simulation for suturing, reducing surgical training costs and time.
Cheng et al., 2021 [49]	AI-based Automated Laparoscopic Cholecystectomy Analysis	Cohort Study	China (Multiple Centers)	The deep learning model identified surgical phases with high accuracy; accuracy varied with case complexity.
Stefanidis et al., 2009 [50]	Initial Laparoscopic Basic Skills Training Shortens the Learning Curve of Laparoscopic Suturing and Is Cost-Effective	Randomized Controlled Trial (RCT)	USA	Basic laparoscopic skills training before suturing reduces learning time and cost. Group I (basic skills training) had a shorter suturing learning curve and needed less active instruction. Cost savings of USD 148 per trainee.
Perumalla et al., 2022 [51]	AI-Based Video Segmentation: Procedural Steps or Basic Manoeuvres	Deep Learning Algorithm Study	USA	The AI algorithm achieved 84% accuracy in differentiating basic maneuvers (suturing, knot tying, and suture cutting). Precision: 87.9% for suture throws, 60% for knot ties, and 90.9% for suture cutting. Basic maneuvers aid in error management and skill assessment.
Yang and Shulruf, 2019 [52]	An Expert-Led and Artificial Intelligence System-Assisted Tutoring Course to Improve the Confidence of Chinese Medical Interns in Suturing and Ligature Skills: A Prospective Pilot Study	Pilot Study	China	Compared regular training, expert-led tutoring, and expert-led + AI tutoring, with the AI-integrated group showing the best OSCE performance and confidence, especially after three AI sessions, underscoring AI’s value in surgical education.
Sirajudeen et al., 2024 [53]	Deep Learning Prediction of Errors in Robotic Suturing	Observational Study	UK	The AI model predicted surgical errors with strong correlations to human assessments in robotic prostatectomy.
Levin et al., 2024 [54]	Automated Identification of Key Steps in Hysterectomy	Retrospective Study	Israel (Five Medical Centers)	AI accurately identified surgical steps in hysterectomy with high precision; further research is needed for clinical application.

## Data Availability

The original contributions presented in this study are included in the article. Further inquiries can be directed to the corresponding author(s).

## Abbreviations

AI	Artificial Intelligence
DTW	Dynamic Time Warping
FP	Future Potential
FST	Future Surgeons Training
LST	Laparoscopic Suturing Training
LR	Large-Scale Research
LE	Lasting Retention of Essential Techniques
ML	Machine Learning
MU	Multi-Center Studies
OSATS	Objective Structured Assessment of Technical Skills
PA	Procedural Accuracy
RC	Resource-Constrained
RCT	Randomized Controlled Trial
RTC	Real-Time Feedback and Correction
SR	Skill Retention
STP	Short-Term Performance
SSE	Sustainable Surgical Education
SS	Surgical Skills
LTP	Long-Term Performance
VR	Virtual Reality
